# Supplementary material for: Remediation of Acid Mine Drainage (AMD) Using Steel Slag: Mechanism of the Alkalinity Decayed Process
Source: Int J Environ Res Public Health. 2023 Feb 4;20(4):2805. doi: 10.3390/ijerph20042805 (PMC9956301; doi:10.3390/ijerph20042805)
Supplement: Supplementary file 1 [file ijerph-20-02805-s001.zip › ijerph-2057643-supplementary.pdf]

Table S1. Utilization ratios of steel slags in different countries (%).

Application	Inner recycling	Road	Cement	Construction	Temporary storage	Others	Unutilized
Japan	20.8	32.4	3.4	34.8	-	7	1.6
USA	-	49.7	3.3	16	-	15.4	15.6
China	7.5	2.6	9.3	-	-	4.7	70
Europe	11	43	5	6	19	3	13
